# Oxidative modification of miR-30c promotes cardiac fibroblast proliferation via CDKN2C mismatch

**DOI:** 10.1038/s41598-024-63635-2

**Published:** 2024-06-07

**Authors:** Wenguang Chang, Dandan Xiao, Xinyu Fang, Jianxun Wang

**Affiliations:** 1 School of Basic Medical Sciences, College of Medicine, Qingdao University, Qingdao, China; 2 Institute for Translational Medicine, College of Medicine, Qingdao University, Qingdao, China

**Keywords:** Cardiac fibrosis, CDKN2C, miR-30c, O^8^G, Oxidation modification, Cell biology, Molecular biology, Molecular medicine

## Abstract

The response of cardiac fibroblast proliferation to detrimental stimuli is one of the main pathological factors causing heart remodeling. Reactive oxygen species (ROS) mediate the proliferation of cardiac fibroblasts. However, the exact molecular mechanism remains unclear. In vivo, we examined the oxidative modification of miRNAs with miRNA immunoprecipitation with O^8^G in animal models of cardiac fibrosis induced by Ang II injection or ischemia‒reperfusion injury. Furthermore, in vitro, we constructed oxidation-modified miR-30c and investigated its effects on the proliferation of cardiac fibroblasts. Additionally, luciferase reporter assays were used to identify the target of oxidized miR-30c. We found that miR-30c oxidation was modified by Ang II and PDGF treatment and mediated by excess ROS. We demonstrated that oxidative modification of G to O^8^G occurred at positions 4 and 5 of the 5′ end of miR-30c (4,5-oxo-miR-30c), and this modification promoted cardiac fibroblast proliferation. Furthermore, CDKN2C is a negative regulator of cardiac fibroblast proliferation. 4,5-oxo-miR-30c misrecognizes CDKN2C mRNA, resulting in a reduction in protein expression. Oxidized miR-30c promotes cardiac fibroblast proliferation by mismatch mRNA of CDKN2C.

## Introduction

Cardiac fibrosis caused by various cardiac diseases leads to increased stiffness and eventually diastolic and systolic cardiac dysfunction. The pathological factors of cardiac fibrosis are complex and involve abnormal proliferation of cardiac fibroblasts and deposits of extracellular matrix proteins and collagens^1^. Reactive oxygen species (ROS) promote the proliferation of cardiac fibroblasts. Mouse hearts treated with MitoParaquat, a mitochondrial ROS generator, exhibited increased cardiac fibroblast proliferation^2^. Although ROS decreased with superoxide dismutase mimetics (Tempol and EUK-8), collagen production in fibroblasts was reduced by Ang II treatment^3^. EUK-8 also attenuated cardiac fibrosis in a pressure-overloaded mouse model^4^. ROS play a vital role in the proliferation and accumulation of cardiac fibroblasts. However, it is unclear exactly what molecular mechanisms ROS use to induce cardiac fibrosis.

With the growing interest in noncoding RNAs and their functions, miRNAs have been widely investigated and are considered potential targets and modulators of oxidative stress-related pathways. MiRNAs are types of noncoding RNAs that are 21–24 nucleotides in length, and the posttranslational regulatory effects of miRNAs have been widely investigated worldwide. Many miRNAs, such as miR-125b^5^, miR-29b^6^, miR-30c^7^ and miR-133^8^, were reported to be correlated with cardiac fibrosis. On the one hand, some miRNAs promote the expression of genes related to fibrosis and facilitate angiogenesis in cardiac tissue. Modifications of certain miRNAs have been shown to be key regulators of cardiac cell functions^9^.

The oxidative modification of the nucleotide changes guanosine (G) to 8-oxoguanosine (O^8^G), which can pair with adenosine (A) and induce guanosine-to-thymidine (G>T) mutations in DNA. Similarly, guanosine in the RNA chain was also found to be modified to O^8^G. Our group previously found that oxidative modified miR-184 exerts its pro-apoptotic effects by targeting the anti-apoptotic proteins BCL-XL and BCL-W by mismatching G to A^9^. Furthermore, a recent report showed that the cardiac hypertrophy inducer phenylephrine induces O^8^G modification at position 7 of miR-1, and this modification is responsible for cardiac hypertrophy phenotypic alteration^10^. Whether oxidative modification also plays an important role in the process of cardiac fibroblast proliferation has not been reported.

Our present work demonstrated that oxidative modification of guanosine (G) to 8-oxoguanosine (O^8^G) occurred in miR-30c and participated in the cardiac fibroblast proliferation process. MiR-30c after oxidative modification misrecognizes CDKN2C, resulting in its reduction, which is responsible for the proliferation and accumulation of cardiac fibroblasts. Our research enriches the understanding of the role of miRNA oxidative modification in cardiac fibrosis development by mispairing with its nonnative targets.

## Methods

### Animal models

All experimental procedures were approved by the Ethics Committee of Qingdao University Medical College. To induce cardiac fibrosis, (1) Osmotic minipumps (Alzet, Cupertino, California, USA) were implanted in mice (4 weeks old) subcutaneously along the animal's dorsum under aseptic conditions to deliver angiotensin II (Ang II, 1500 ng/kg/min per mouse, Sigma, China) or vehicle (0.01 N acetic acid in saline) for 28 days as previously described^11^ with minor modifications. (2) The hearts of 8-week-old adult male C57BL/6 mice were subjected to 45 min of ischemia followed by 3 h of reperfusion as previously described^12^. Heart tissues were collected for total miRNA extraction and dot blotting.

### Cell culture and drug treatment

Neonatal rat cardiac fibroblasts (NRCFs) were obtained from Sprague‒Dawley rats (1–3 days) as described^13^. Custom synthesis and modification services from Takara Biotechnologies (Japan) were used to produce RNAs with various modifications, of which the quality was monitored, reported and confirmed by the company through mass spectrometry. 8-oxoguanine (O^8^G) in the ribonucleotide was introduced at the indicated position of 3-oxi-miR-30c (5′-CUO^8^GGGAGAAGGCUGUUUACUCU-3′); 4-oxi-miR-30c (5′-CUGO^8^GGAGAAGGCUGUUUACUCU-3′); 5-oxi-miR-30c (5′-CUGGO^8^GAGAAGGCUGUUUACUCU-3′); and 4,5-oxi-miR-30c (5′-CUGO^8^GO^8^GAGAAGGCUGUUUACUCU-3′). MiR-30c containing a substitution of G in the indicated position with U was also synthesized (4,5-U-miR-30, 5′-CUGUUAGAAGGCUGUUUACUCU-3′) and duplexed with the passenger strand of miR-30c. As a control miRNA, nontargeting miRNA (NC) was synthesized as siRNA and used as a duplex (sense strand: 5′-UUCUCCGAACGUGUCACGUTT-3′, antisense strand: 5′-ACGUGACACGUUCGGAGAATT-3′). Cells were transfected with miRNA duplexes (50 nM) or antagomir (50 nM) using Lipofectamine 3000 (Invitrogen) according to the manufacturer’s instructions.

### Fenton reaction and oxidized miR-30c extraction

Mir-30c mimic (sense strand: 5′-CUGGGAGAAGGCUGUUUACUCU-3′, antisense strand: 3′-AGUAAACAGCCUUCUCCCAGUU-5′) was custom synthesized by Takara Biotechnologies (Japan). The Fenton reaction was conducted by using synthesized miR-30c. Briefly, miR-30c (80 mg) was incubated with 2 mM H_2_O_2_ using redox cycling with ferric iron (Fe^3+^, 0.5 mM) or cupric copper (Cu^2+^, 0.5 mM) along with the reducing agent ascorbate (Asc, 5 mM) in 10 mM NaH_2_PO_4_/Na_2_HPO_4_ buffer (pH 7.4). The samples were incubated at 37 °C for 1 h, and the reactions were terminated by adding 10 mM of the metal chelators DFOM (for Fe^3+^) or cuprizone (for Cu^2+^) and placing the samples on ice. The oxidized miR-30c was precipitated in 3 M ice-cold sodium acetate (pH 5.0) and 2.5 volumes of ethanol, centrifuged, washed in 70% V/V ethanol and hydrolyzed.

### miRNA extraction

To purify RNA according to size, the miRNeasy Mini Kit (Qiagen) was used to separate small (< 200 nt) and large (> 200 nt) RNA fractions, following the protocol provided by the manufacturer. For heart tissue samples, a Minilys Personal Homogenizer (Bertin) or glass homogenizer was used in the presence of 700 μl Qiazol Lysis Reagent (Qiagen) supplemented with 2.5 mM DFOM (Sigma‒Aldrich) to prevent in vitro oxidation as previously reported. For total RNA purification, only Qiazol Lysis Reagent with 2.5 mM DFOM was used with the addition of 20% chloroform (Merck), followed by RNA precipitation with isopropyl alcohol and treatment with RQ1-free DNase (37 °C for 30 min; Stop solution with heat inactivation, 65 °C for 10 min; Promega). For the precise quantitation of RNA, both a spectrophotometer (Denovix) and Qubit fluorometric quantification (Invitrogen) were used. To purify miRNA, 20 nt total RNA gel extraction of ~ 20 nt from total RNA was performed. Total RNA denatured in Gel Loading Buffer II (at 90 °C for 2 min; Ambion) was separated by 15% Urea-PAGE gel with the indication of ~ 20 nt miRNA marker (synthesized miR-1), of which the size was cross-checked with 14–30 ssRNA ladder marker (Takara). After staining with SYBR Gold (Invitrogen), an ~ 20 nt fragment in the gel was excised, incubated with gel extraction buffer (0.5 M ammonium acetate, 10 mM magnesium acetate, 0.1% SDS, 1 mM EDTA, pH 8.0) at 65 °C overnight in a thermomixer (Thriller, Peqlab), and further purified by an Oligo Clean & Concentrator Kit (Zymo).

### Dot blotting analyses

For dot blotting, size fractioned RNA (80–300 ng) was dotted on the Zeta-Probe blot membrane (Bio-Rad) and crosslinked by UV irradiation with the option of optimal crosslinking (120 mJ/cm^2^) in Spectrolinker XL-1000 (Spectroline). The membrane was blocked with 1 × TBST with 5% BSA for 1 h at RT and incubated with anti-O^8^G antibody (15A3, 1:2000; QED Bioscience) in 1 × TBST for 1 h at RT. HRP-conjugated goat anti-mouse IgG (1:2000, Abcam, Shanghai, China) was then used in TBST for 1 h at RT, followed by reaction with ECL (Vazyme, Nanjing, China) for detection. The blot signal was calculated as the relative intensity of the total amount of miRNA, stained by SYBR Gold (1:10,000, Invitrogen).

### *Immunoprecipitation of miRNAs by O*^*8*^*G*

Immunoprecipitation (IP) of O^8^G with an anti-O^8^G antibody (15A3, QED Bioscience) was performed as previously reported with a high-detergent IP buffer and washed with high-salt conditions as indicated^10^. RNA in the bead was purified by 700 μl of Qiazol Lysis Reagent supplemented with 2.5 mM DFOM and 20% chloroform, followed by the RNA Clean & Concentrator-5 kit (Zymo).

### Quantitative RT-PCR for miRNAs

To quantify miRNA, we used the miRNA qPCR method as previously described. Briefly, 1 μg of small RNA was purified by the miRNeasy Mini Kit (Qiagen), and qPCR was performed in SYBR Green PCR Master Mix (Vazyme, Nanjing, China) with forward primer (the same sequence as miRNA) and reverse primer (5′-CCAGTGCAGGGTCCGAGGT-3′); cycling conditions were as follows: 5 min at 95 °C; 45 cycles of 15 s at 95 °C, 15 s at 55 °C and 20 s at 72 °C; and 5 min at 72 °C. U6 snRNA was measured as a reference control in general (forward: 5′-ATTGGAACGATACAGAGAAGATT-3′, reverse: 5′-GGAACGCTTCACGAATTTG-3′).

### *Quantification of oxidized miRNA by O*^*8*^*G IP and qPCR*

After NRCFs were treated with H_2_O_2_ for 24 h or Ang II (50 nM) or PDGF (50 ng/L), O^8^G immunoprecipitation was performed as previously described^10^. Briefly, small RNA was extracted using the miRNeasy Mini Kit (Qiagen). The same amount of small RNA (2 μg), precisely measured by Qubit fluorometric quantification (Invitrogen), was then used for O^8^G immunoprecipitation. After extracting RNA from the bead using the Qiazol Lysis Reagent and RNA Clean and Concentrator-5 kit (Zymo), the amount of a specific miRNA in O^8^G IP was evaluated using the TaqMan MicroRNA Assay kit (Applied Biosystems). In addition, the relative level was also estimated by measuring the specific miRNA in the input and was used for normalization to confirm the enrichment of oxidation in a specific miRNA.

### RNA extraction and quantitative real-time PCR

Total RNA from NRCFs was extracted using TRIzol RNA isolation reagent (Invitrogen). RNAs were reverse transcribed to cDNA using TransScript II First-Strand cDNA Synthesis SuperMix (Vazyme). Quantitative real-time PCR was performed using SYBR Master Mix (Vazyme). Fold changes in gene expression were calculated using the 2^–ΔΔ^Ct method, and the expression of GAPDH was used as an internal control. The qPCR primers were as follows: TGF-β1 (forward: CCAGATCCTGTCCAAACTAAGG; reverse: CTCTTTAGCATAGTAGTCCGCT); FN-1 (forward: ACCCTTCCACACCCCAATC, reverse: TTGCCCAACACTGGGTTGTT); POSTN (forward: AAGACCACACAGGGAAGCAAA, reverse: AACCGGAATGTCTGCTGGAT).

### Luciferase reporter assay

To measure the activity of oxidized miRNA-mediated gene repression, psiCheck-2 (Promega) was used. In particular, for the miR-30c oxo sites, the target sites were repeated in tandem (n = 5) and inserted into the 3′UTR of Renilla luciferase (hRLuc) of psiCheck-2 to increase detection sensitivity. For this vector construction, synthetic duplex oligos (TsingKe) containing various miR-30c 4,5-oxo sites were cloned and inserted into the psiCheck-2 plasmid as indicated: 4,5-oxo-miR-30c target site, 5′-GCCTTCTAACAGCTCTAACAGCTCTAACAGCTCTAACAGCTCTAACAGCGGC-3′; 4,5oxo-mut sites: 5′-GCCTACTTTGAGCACTTTGAGCACTTTGAGCACTTTGAGCACTTTGAGCGGC-3′.

To generate reporter vectors with binding sites for 4,5-oxo-miR-30c, the 3′UTR of CDKN2C was cloned and inserted into the pGL3 vector (Promega) immediately downstream of the stop codon of the luciferase gene. For the luciferase assay, cells in 24-well plates were co-transfected with 200 ng per well of the plasmid construct pGL3-CDKN2C-3′UTR and 400 ng per well of 4,5-oxo-miR-30c, 4,5-mut-miR-30c or miR-30c using Lipofectamine 3000 (Invitrogen). The pRL-TK vector containing Renilla luciferase cDNA (5 ng per well) served as an internal control, and 4,5-mut-miR-30c or miR-30c served as a negative control. Twenty-four hours after transfection, the cells were lysed, and luciferase activity was measured with a dual luciferase kit (Promega).

### Western blot analysis

For in vitro Western blot experiments, after completion of the experiments, cardiomyoblast cells were harvested using ice-cold RIPA lysis buffer. Protein concentrations were measured using the Bradford assay (Bio-Rad protein assay kit). For the detection by western blot of Col 1A, MMP2, CDKN2C, CDK4, cyclin D, MYBL2 and β-actin expression, the method used was the same as that described previously. Proteins for in vivo and in vitro experiments were visualized with enhanced chemiluminescence solution, and images were generated with a GENE imaging system. Images were quantified using Image Analysis Software (Quantity One). Primary antibodies against Col1A (catalog number, A16891), MMP2 (catalog number, A19080), and CDK4 (catalog number, A0366) were purchased from ABclonal (Wuhan, China). MYBL2 (catalog number, BA3860) was purchased from Boster Biological Technology (USA). CDKN2C (catalog number, AF5356) was purchased from Affinity Bioscience (USA). β-Actin and HRP-conjugated secondary antibodies were purchased from Yesen Biotechnology (Shanghai, China).

### Statistical analysis

Data are presented as the mean ± SEM. An unpaired two-tailed Student’s t test was used to determine significant differences between groups. The adjusted two-sided P values were calculated, and P < 0.05 was considered indicative of statistical significance.

### Ethics approval

All methods were carried out in accordance with relevant guidelines and regulations and was approved by the Animal Ethics Committee of Qingdao University. The study is reported in accordance with ARRIVE guidelines.

## Results

### The miRNAs were oxidatively modified in the hearts of injured mice

**Figure 1:**
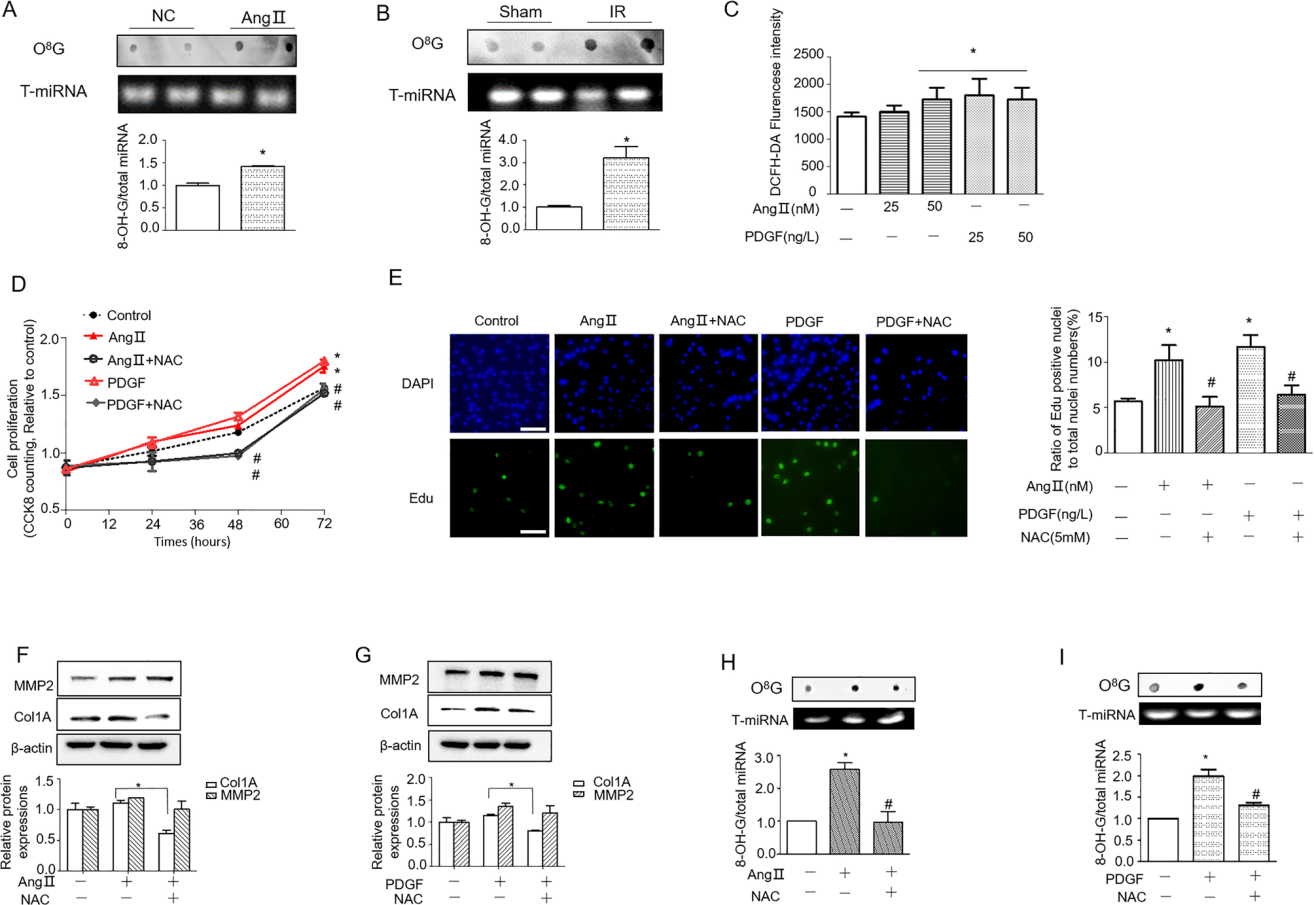
O^8^G modification of miRNA is found in vivo and in vitro. (**A**,**B**) Dot blotting analysis of O^8^G in small RNAs from the hearts of (**A**) Ang II-induced heart injury animals, *p < 0.05 compared with the NC group; (**B**) I/R-induced heart injury animals. *p < 0.05 compared to the sham group. (**C**) ROS measurements in NRCFs treated with Ang II or PDGF. (**D**) Cell proliferation in NRCFs treated with Ang II/PDGF with or without NAC. (**E**) EdU labeling of synthesized DNA to show the proliferation of NRCFs treated with Ang II/PDGF with or without NAC. Scale bar, 100 μm. (**F**,**G**), Representative Western blot (top) and densitometric analysis (bottom) of MMP2 and Col1A protein levels in NRCFs treated with AngII (**F**) or PDGF (**G**). Dot blot analysis of O^8^G in small RNAs from NRCFs treated with AngII (**H**) or PDGF (**I**). Total small RNAs were used as a loading control. *p < 0.05 compared to the control group, ^#^p < 0.05 compared to the group treated with AngII or PDGF.

First, we investigated whether miRNAs were oxidized in the hearts of injured mice. In our experiments, cardiac fibrosis models were chosen. Mice were subjected to myocardial infarction or implanted minipumps loaded with Ang II (1.5 µg/kg/min) as described in the methods. Then, total miRNAs were extracted with a miRNA kit from the heart tissues of mice. Dot blotting was used to detect the presence of oxidized guanosine (O^8^G) in miRNAs. The results showed that the miRNAs with O^8^G modification increased significantly in both hearts with Ang II treatment and hearts with myocardial infarction injury (Fig. 1A,B).

Ang II and PDGF are two well-known agents that can promote fibroblast proliferation in vitro. Incubation of Ang II (25 nM, 50 nM) or PDGF (25 ng/L, 50 ng/L) with neonatal primary CFs was shown to increase the intensity of DCFH-DA fluorescence, which is an indicator of ROS generation (Fig. 1C). Further, we used N-acetylcysteine (NAC) as an antioxidant to observe cell proliferation and the oxidation of miRNAs after treatment with Ang II and PDGF. Treatment with Ang II and PDGF promotes cardiac fibroblast proliferation. However, treatment with the antioxidant NAC decreased the proliferation-promoting effects induced by Ang II and PDGF (Fig. 1D). Indicating that the oxidative modification of O^8^G is associated with excessive oxidative stress. These results were further confirmed by EdU labeling experiments, which also demonstrated that NAC treatment decreased AngII- and PDGF-induced fibroblast proliferation (Fig. 1E). Concomitantly, NAC significantly reduced Ang II- and PDGF-induced Col1A elevation, which is a cardiac fibrosis biomarker (Fig. 1F,G, indicating that oxidative stress participates in the process of myocardial fibrosis. Furthermore, oxidized miRNAs were also detected in Ang II-treated and PDGF-treated CFs. Treatment with NAC decreased oxidized miRNA expression in cells treated with AngII and PDGF (Fig. 1H,I).

### MiR-30c was oxidized in AngII- and PDGF-treated cardiac fibroblasts

**Figure 2:**
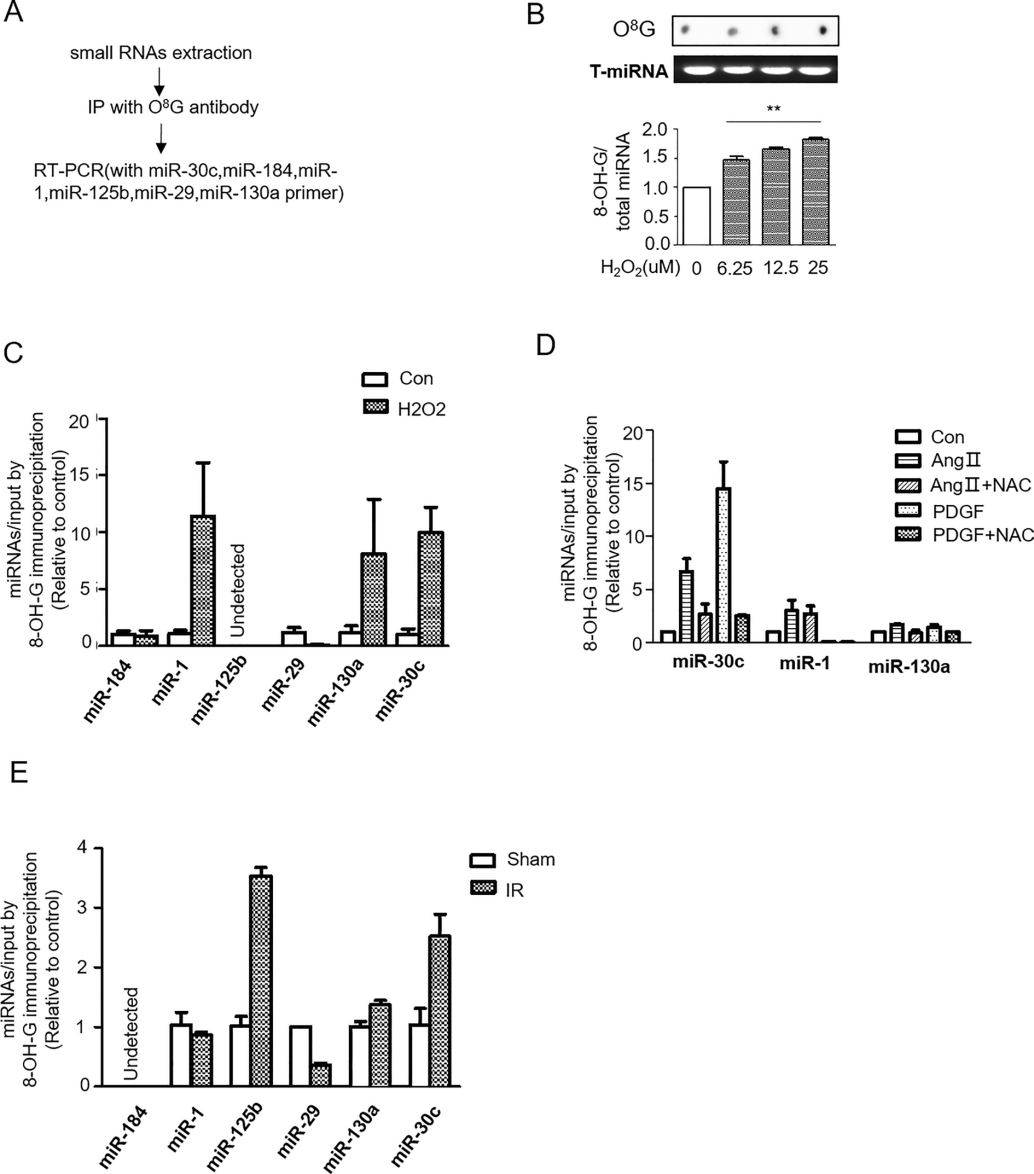
MiR-30c is oxidized by Ang II or PDGF treatment. (**A**) Schematic of the O^8^G miRNA identification process. (**B**) O^8^G dot blotting analysis of small RNAs from H_2_O_2_-treated NRCFs. **p < 0.01 compared to the control group. (**C**) Oxidized miRNA expression in NRCFs by H_2_O_2_ treatment. (**D**) Oxidized miRNA expression in NRCFs treated with Ang II or PDGF with or without NAC. (**E**) Oxidized miRNA expression in heart tissues from sham and IR mice. *p < 0.05 compared to the control group, ^#^p < 0.05 compared to the group treated with AngII or PDGF.

Several miRNAs were shown to participate in the progression of myocardial fibrosis. For example, oxidized miR-1 has been reported to cause cardiac hypertrophy in the hearts of isoproterenol-treated mice. Because the oxidized ability of AngII and PDGF was mild, in our experiments, we use hydrogen peroxide (H_2_O_2_) as a strong oxidant to demonstrate that miRNAs can undergo oxidative modification (Fig. 2A,B). Then, we conducted a literature search and identified several miRNAs (such as miR-1, miR-125b, miR-184, miR-30c, and miR-130a) that have been proven to be closely associated with fibroblast proliferation. And using the RNA immunoprecipitation method, we screened some miRNAs (including miR-1, miR-30c, and miR-130a) that were oxidized by H_2_O_2_ treatment in CFs (Fig. 2C). Furthermore, in the following experiments, we use Ang II and PDGF, which are factors that have been proven by many studies to promote myocardial fibrosis, to demonstrate the role of miRNA oxidative modification in the process of myocardial fibrosis. We detected that if the selected three miRNAs were also modified by AngII and PDGF treatment, the results showed that miR-30c has a high positive reaction for the anti-O^8^G antibody by AngII and PDGF treatment, and NAC treatment reduces the IP of the anti-O^8^G antibody with miR-30c (Fig. 2D). In addition, we confirmed the elevation of oxidized miR-30c in the heart tissue of the IR group (Fig. 2E). These results indicate that oxidized miR-30c may play a role in AngII- and PDGF-induced cell proliferation.

### Oxidized miR-30c promotes the proliferation of CFs

**Figure 3:**
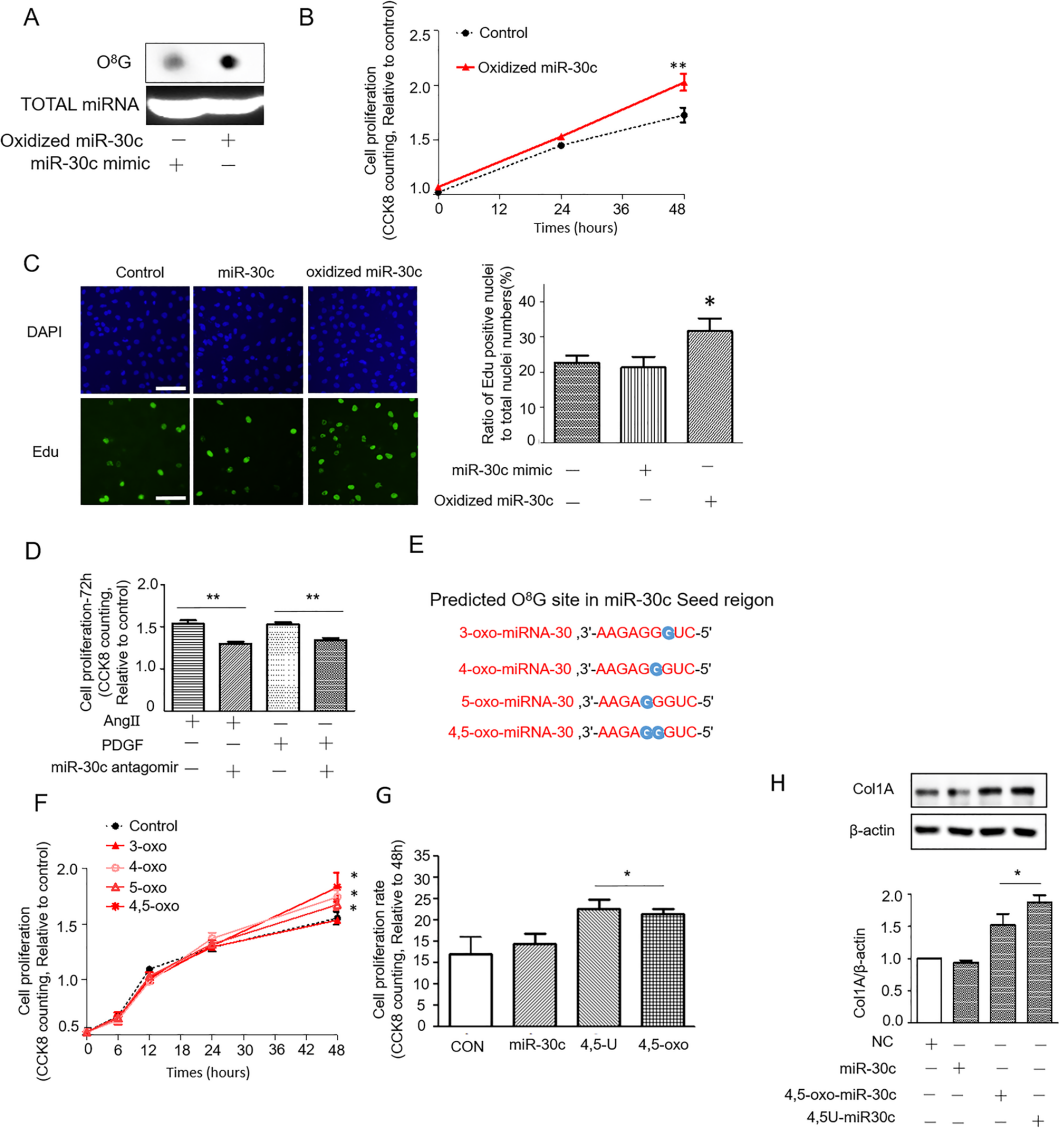
Oxidized miR-30c participates in AngII- or PDGF-induced cardiac fibrosis. (**A**) Dot blotting analysis of O^8^G in oxidized miR-Ang II-30c by the Fenton reaction. (**B**) Cell proliferation in NRCFs treated with miR-30c or oxidized miR-30c. (**C**) EdU labeling of synthesized DNA to show the proliferation of NRCFs treated with miR-30c or oxidized miR-30. Scale bar, 100 μm. (**D**) Cell proliferation in NRCFs treated with Ang II/PDGF with or without miR-30c antagomir. (**E**) Predicted O^8^G site in the miR-30c seed region. (**F**) Cell proliferation in NRCFs treated with 3-oxo, 4-oxo, 5-oxo, and 4,5-oxo-miR-30c. (**G**), Cell proliferation (48 h) in NRCFs treated with miR-30c, 4,5-U-miR-30c, and 4,5-oxo-miR-30c. (**H**), Representative Western blot (top) and densitometric analysis (bottom) of Col1A protein levels in NRCFs treated with miR-30c, 4,5-U-miR-30c, and 4,5-oxo-miR-30c. *p < 0.05 compared to the control group.

Furthermore, to investigate the role of oxidized miR-30c, we first oxidized the synthesized miR-30c by the Fenton reaction in vitro as described in the Methods. Then, oxidized miR-30c was added to the CFs. The guanine of miR-30c was significantly oxidized by the Fenton reaction in vitro (Fig. 3A), which was confirmed by dot blotting with the O^8^G antibody. Treatment of CFs with oxidized miR-30c (oxi-miR-30c) significantly increased proliferation (Fig. 3B). This result was further confirmed by EdU labeling experiments (Fig. 3C). The miR-30c antagomir reduced AngII- and PDGF-induced proliferation promotion effects (Fig. 3D).

Motivated by the detection of O^8^G-miR-30c, we further examined the oxidized G positions in miR-30c seed regions. Previous studies have shown that the repeated G sequence is more susceptible to oxidation compared to a single G due to its lower redox potential^14,15^. In our study, the miR-30c sequence has three continuous G at the end of the 5′ in the seed region; thus we aim to further confirm the oxidized position (whether the oxidized G was at positions 3, 4 or 5) (Fig. 3E). By a chemical modification method, we synthesized O^8^G-modified miR-30c at the indicated seed position (denoted 3-oxo-miR-30c, 4-oxo-miR-30c, 5-oxo-miR-30c, and 4,5-oxo-miR-30c) and used those modified miR-30c to incubate with CFs. The results showed a significant promotion by these synthesized miRNAs as early as 48 h (Fig. 3F) compared to 72 h by AngII and PDGF treatment alone in CFs (Fig. 1D). Specifically, the proliferation of CFs induced by 4,5-oxo-miR-30c had the highest significance. This effect was found to depend on the pairing of O^8^G • A bases because the substitution of O^8^G with U (forming 4,5U-miR-30c) could induce a similar proliferation effect (Fig. 3G). Furthermore, 4,5-oxo-miR-30c and 4,5-U-miR-30c substantially increased Col1A expression (Fig. 3H). The fibrosis-promoting effects of oxidized miR-30c in CFs.

### *4, 5-oxo-miR-30c binds to the targeted mRNA by pairing the O*^*8*^*G ⋅ A base*

**Figure 4:**
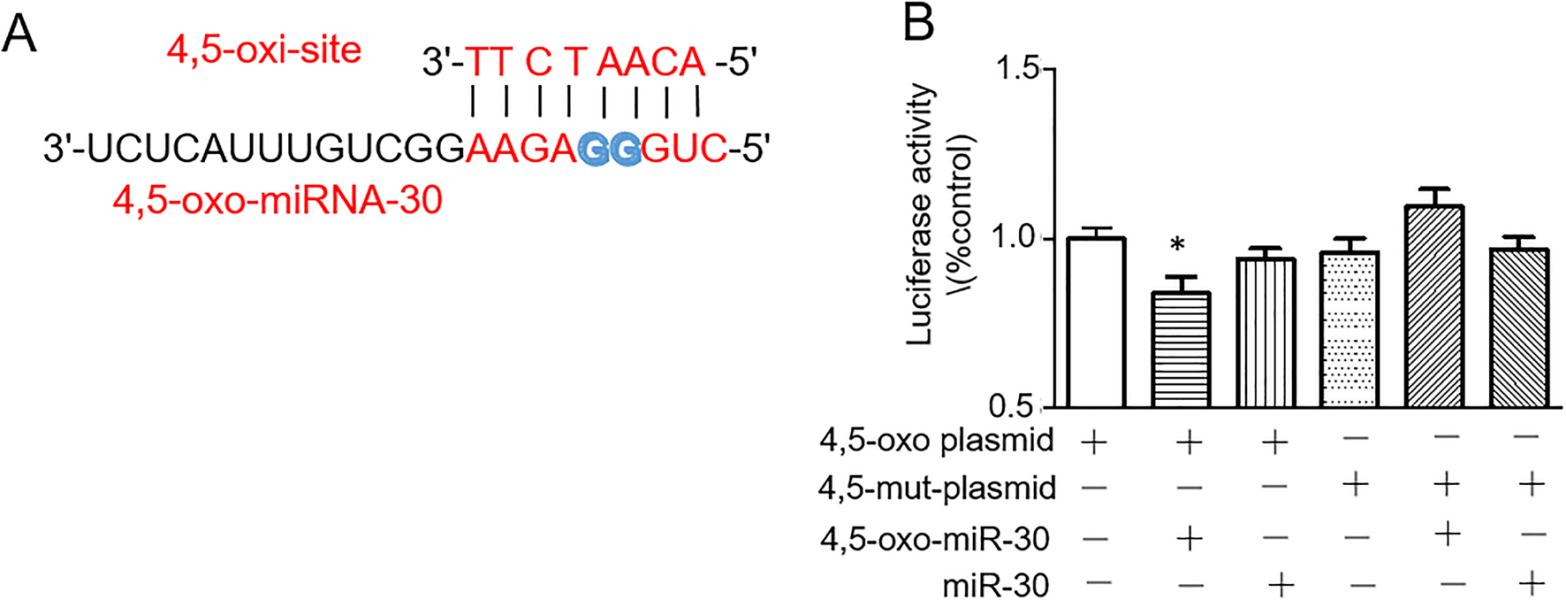
4,5-oxo-miR-30 redirects target repression via O^8^G • A base pairing. (**A**) Sequence of 4,5-oxo-miR-30c and its predicted target sites in the seed region. (**B**) Luciferase reporter assays for 4,5-oxo sites using synthesized 4,5-oxo-miR-30c and miR-30c. Activity is reported relative to the nontargeting control. *P < 0.05.

To further confirm the mismatch of O^8^G with A. Firstly, we constructed a luciferase reporter plasmid with repeated base sequences complementary to the seed regions of 4, 5-oxo-miR-30c, the complementary CC bases at positions 4 and 5 were substituted with AA in the repeated sequence (as described in the Methods) (Fig. 4A). If the results showed 4, 5-oxo-miR-30c can pair with the sequence and reduce the luciferase activity, it indicates that the GG sites of 4, 5-oxo-miR-30c can pair with the AA site. Luciferase reporter assays demonstrated that 4,5-oxo-miR-30c could silence targets through cognate oxo sites by decreasing the luciferase activity. In contrast, miR-30c mimic or 4,5-mut-miR-30c did not exhibit similar effects(Fig. 4B).

### CDKN2C is the target of 4,5-oxo-miR-30c

**Figure 5:**
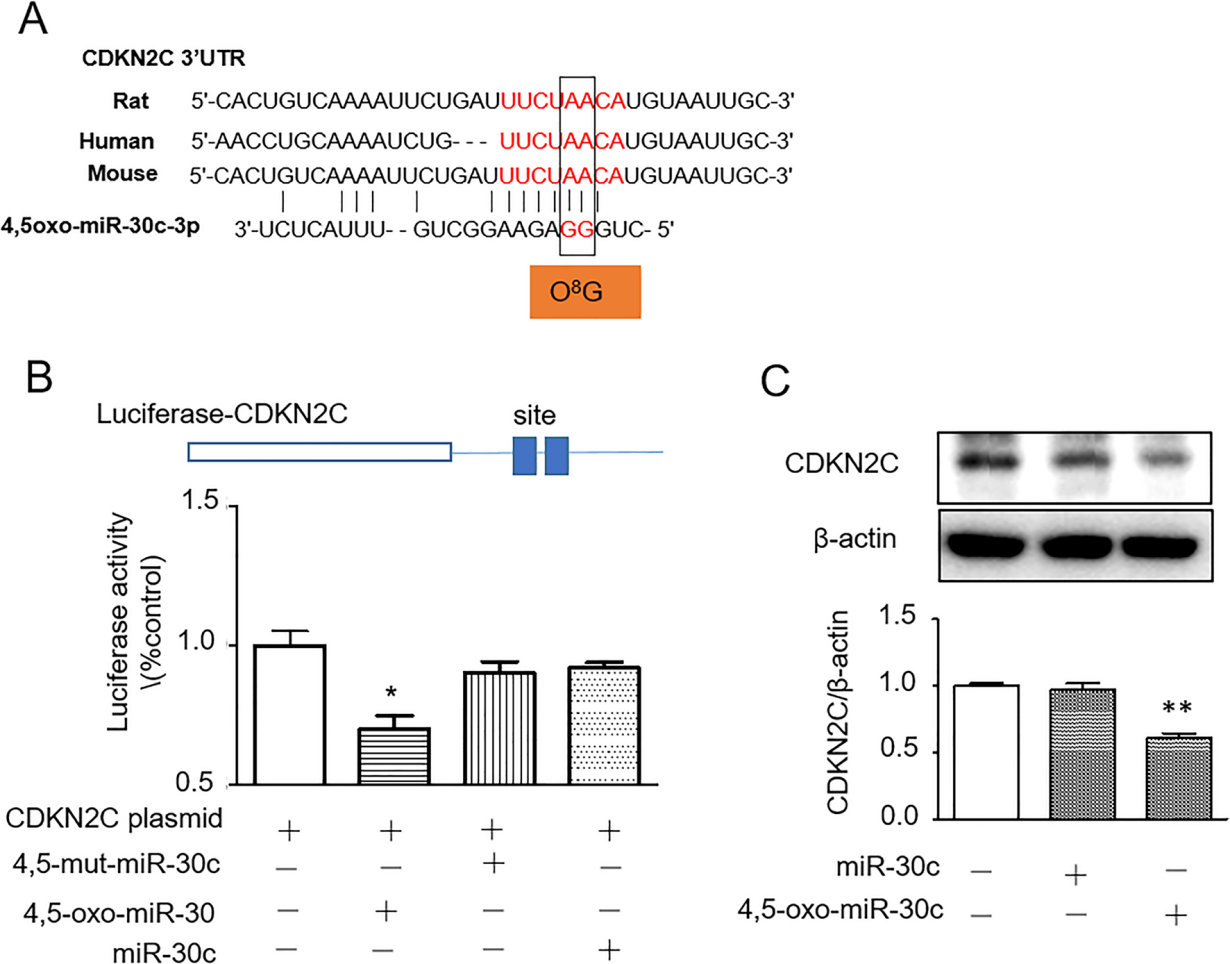
CDKN2C is the target of 4,5-oxo-miR-30c. (**A**) 4,5-oxo-miR-30c and homologies of its predicted target sites in the 3′UTR of CDKN2C mRNA. (**B**) Luciferase reporter assays for the 3′UTR of CDKN2C mRNA using synthesized 4,5-oxo-miR-30c, 4,5-mut-miR-30c, and miR-30c. Activity is reported relative to the nontarget control. *P < 0.05. (**C**) Representative Western blot (top) and densitometric analysis (bottom) of CDKN2C protein levels in miR-30c and 4,5-oxo-miR-30c NRCFs. *p < 0.05 compared to the control group.

Investigate the underlying mechanism of the effects of 4,5-oxo-miR-30c on cell proliferation. We searched the miRDB website in a custom prediction tab (species: mouse, submission type: miRNA sequence (CUGUUAGAAGGCUGUUUACUCU)) and found that 4,5-oxo-miR-30c (with mismatched O^8^G with A) may have a good binding site (miRDB: target rank: 6; target score: 93) with CDKN2C mRNA (Fig. 5A). We constructed a luciferase reporter plasmid with the 3′-UTR of CDKN2C and then transfected the plasmid with 4,5-oxo-miR-30c, 4,5-mut-miR-30c or miR-30c into CFs. In this study, if 4, 5-oxo-miR-30c can target CDKN2C by pairing its 3′-UTR with mismatching GG with AA sites, suggesting 4, 5-oxo-miR-30c can regulate CDKN2C expression by mismatching oxidized GG with AA in the 3′UTR of CDKN2C. The results showed that 4,5-oxo-miR-30c could reduce luciferase activity by binding to the CDKN2C 3′UTR, but miR-30c mimic or 4,5-mut-miR-30c had no such effects (Fig. 5B). We confirmed the results with the CDKN2C antibody, which showed that compared to miR-30c treatment, 4,5-oxo-miR-30c significantly decreased the expression of CDKN2C in CF (Fig. 5C). Additionally, as MYBL2 is a known target for miR-30c, we confirmed the results with the MYBL2 antibody, which showed that miR-30c treatment significantly decreased the expression of MYBL2 in CF but not by 4,5-oxo-miR-30c (Fig. S1D), indicating that oxidized miR-30c (4,5-oxo-miR-30c) shifted to regulate the expression of CDKN2C.

### 4,5-oxo-miR-30c targets CDKN2C to regulate cell proliferation

**Figure 6:**
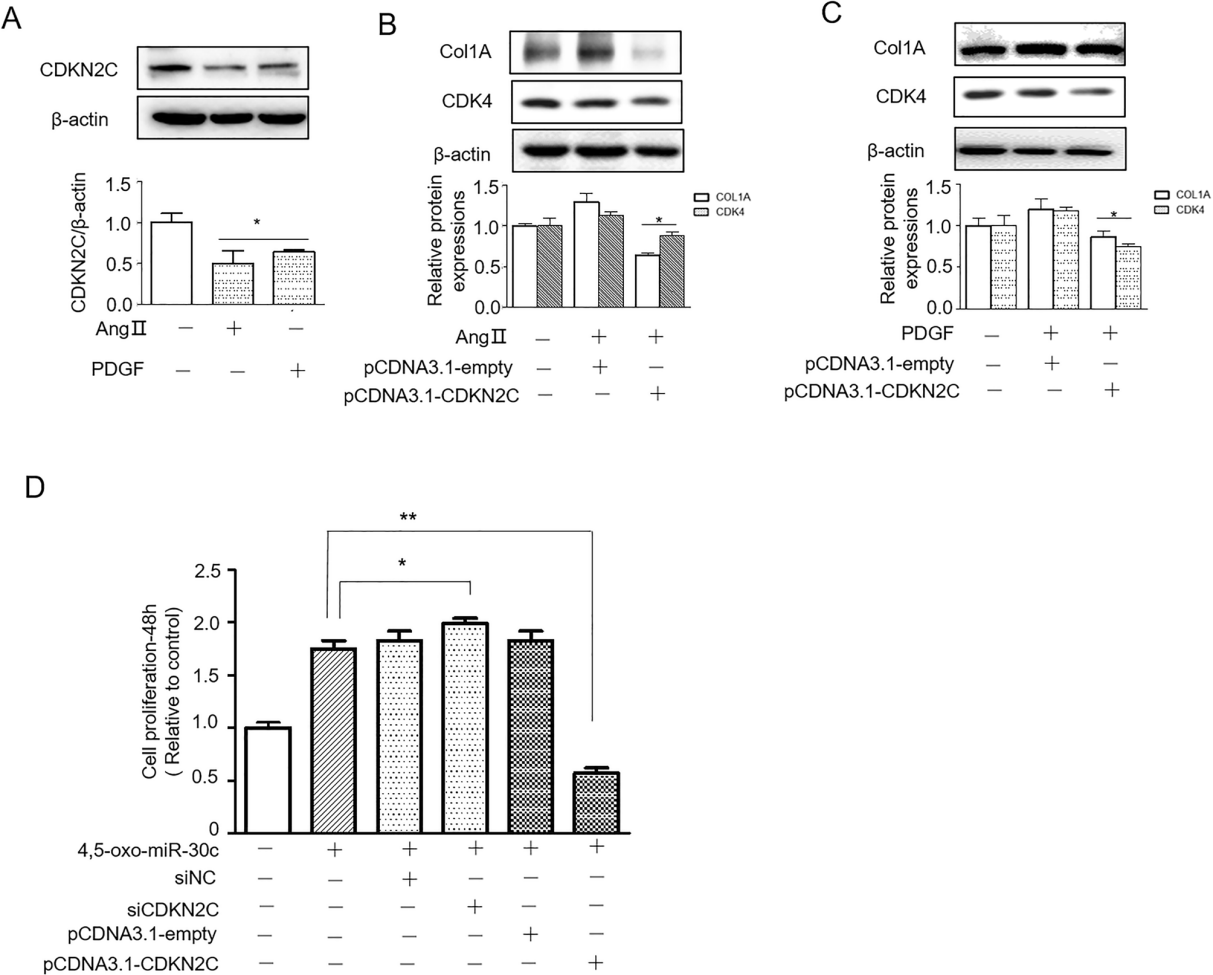
4,5-oxo-miR-30c promotes cell proliferation by targeting CDKN2C in NRCFs. (**A**) Representative Western blot (top) and densitometric analysis (bottom) of CDKN2C protein levels in Ang II/PDGF-treated NRCFs. *p < 0.05 compared to the control group. Representative Western blot (top) and densitometric analysis (bottom) of Col1A protein levels in NRCFs treated with Ang II (**B**) PDGF (**C**) with or without CDKN2C overexpression. *p < 0.05 compared to the control group. (**D**) Cell proliferation (48 h) in NRCFs treated with 4,5-oxo-miR-30c with or without overexpression of CDKN2C or with or without CDKN2C siRNA. *p < 0.05 compared to the control group.

Then, we tested the expression of CDKN2C in CFs by AngII or PDGF treatment; the results showed that the expression of CDKN2C was significantly reduced by both AngII and PDGF treatment (Fig. 6A). In addition, overexpressing CDKN2C diminished ColA expression and the expression of the cell cycle protein CDK4 (Fig. 6B,C). Importantly, 4, 5-oxo-miR-30c promotes cell proliferation, but overexpressing CDKN2C reverses this effects. CDKN2C siRNA has a similar effects to 4, 5-oxo-miR-30c in promoting proliferation (Fig. 6D), this suggests that the proliferation of fibroblast by 4, 5-oxo-miR-30c is at least partly due to the reduction of CDKN2C levels through the 4,5-oxo-miR-30c G mismatch with the A base of the 3′UTR of CDKN2C.

## Discussion

ROS are small reactive molecules containing at least one atom of oxygen with higher reactivity than molecular oxygen, including superoxide anion (O^2−^), hydrogen peroxide (H_2_O_2_), hydroxyl radical (OH^−^), peroxynitrite (ONOO^−^), hypochlorous acid (HOCl), and others^16^. Excess ROS results in cell damage and death, which is called oxidative stress. Oxidative stress is involved in various cardiac diseases, including hypertrophic, fibrotic, and apoptotic cardiac cells^17,18^. Over the last decades, many studies have tried to find the underlying mechanisms of how ROS affect cell functions, but with very limited success. Currently, several pathways and proteins are known to be responsible for ROS action in the heart. For example, ROS are essential for autophagy regulation, dysfunction of which causes cardiac hypertrophy^19^, and ROS-mediated AngII-induced collagen production in cardiac fibroblasts^20^, which is consistent with our results. Reducing ROS by TIM50 overexpression or HIF-1α expression attenuates pathological cardiac hypertrophy^21^ and CF proliferation following myocardial infarction^2^. On the other hand, ROS alter miRNA expression levels and modifications in cells^22,23^. The posttranscriptional modification of RNA can dynamically change the transcriptome, thus modulating gene expression and controlling cellular processes. More than 150 types of modifications were discovered in various types of RNA^24^, including N6-methyladenosine (m6A), N1-methyladenosine (m1 A), 2′-O methylation (2′-O-me), and 5-methylcytosine (m5 C). These modifications of RNAs play an important role in the stabilization of the structure of the RNA, control of RNA processing, and modulation of degradation^25–28^. The 2′-O-me at the 3′ end of miRNAs prevents their degradation^29^, and the modification of lncRNA-XIST by m6A promotes its transcriptional repression^30^. However, the functions of noncoding RNA modifications remain largely unknown.

The 8-oxo-G modification of RNA was first reported in 1989^31^, and this kind of modification was reported to compromise translational activity and inhibit RNA elongation. In addition to acting on the translation process, oxidized RNA could modulate the inflammatory response, because cells transfected with oxidized mitochondrial RNA (mtRNA), isolated from H_2_O_2_-treated HA1 hamster fibroblasts, reduce proinflammatory cytokine production^32^. And the 8-oxo-G modification of miRNA regulates cardiac cell apoptosis by mismatching the mRNA of anti-apoptotic proteins^9^. This suggests that oxidized miRNAs could regulate cellular pathways by targeting certain signals. However, because of the limitations of sequencing technology, the specific oxidation site is difficult to determine. In our study, we identified that miR-30c is oxidized by treatment with Ang II and PDGF in CF. Given that the repeat G sequence is more susceptible to oxidation compared to a single G due to its lower redox potential^14,15,33^, the miR-30c sequence has three continuous G at the end of the 5's in the seed region; thus, we constructed oxidized miR-30c at each position of G separately (3-oxo-miR-30c, 4-oxo-miR-30c, 5-oxo-miR-30c or 4,5-oxo-miR-30c) to identify the precise oxidized position site of miR-30c. The results of our study demonstrated that 4,5-oxo-miR-30c has a similar effect as Ang II or PDGF treatment in CF and binds to the 3′-UTR of CDKN2C mRNA by mismatching G with A, thus promoting CF proliferation. This is the first time that we have found that oxidative modified miRNAs can affect CF proliferation.

Normally, oxidized RNA was reported to bind to AUF1 or PCBP1, and then degradation by exosomes was excluded or by cell apoptosis. AUF1 is capable of interacting with moderately (single 8-oxo G) and severely (8-oxoG × 2) oxidized RNA. PCBP1 can selectively bind to severely oxidized RNA (8-oxoG × 2)^34^. The way in which the degradation process of oxidized miR-30 is determined in our study is not determined in our experiment, which will be our next research direction.

## Conclusions

Above all, our study shows that AngII and PDGF induce excess ROS and lead to O^8^G production in the miR-30c seed region. Therefore, oxidized miR-30c is redirected to repress the new target CDKN2C, which functions in cell cycle pathways. Furthermore, 4,5-G in the miR-30c seed regions was the O^8^G site. Our findings suggest that the formation of O^8^G in miRNA could be at least one of the reasons for the general epitranscriptional mechanism of AngII- and PDGF-induced cardiac fibrosis.

### Supplementary Information

Supplementary Information 1.

Supplementary Information 2.

Supplementary Information 3.

## Data Availability

The datasets used and/or analyzed during the current study available from the corresponding author on reasonable request.

## Abbreviations

AngII: Angiotensin II
AUF1: AU-rich binding factor 1
BCL-xl/w: BCL-2 family members
CDK4: Cyclin D kinase 4
CDKN2C: CDKN2C cyclin dependent kinase inhibitor 2C
CF: Cardiac fibroblast
O^8^G: 8-OH-guanine
PCBP1: Poly(rC)-binding protein 1
PDGF: Platelet derived growth factor
ROS: Reactive oxygen species
3’-UTR: 3’End untranslated region
