# Supplementary material for: Oxidative modification of miR-30c promotes cardiac fibroblast proliferation via CDKN2C mismatch
Source: Sci Rep. 2024 Jun 7;14:13085. doi: 10.1038/s41598-024-63635-2 (PMC11161483; doi:10.1038/s41598-024-63635-2)

Fig.1f,g-mmp2

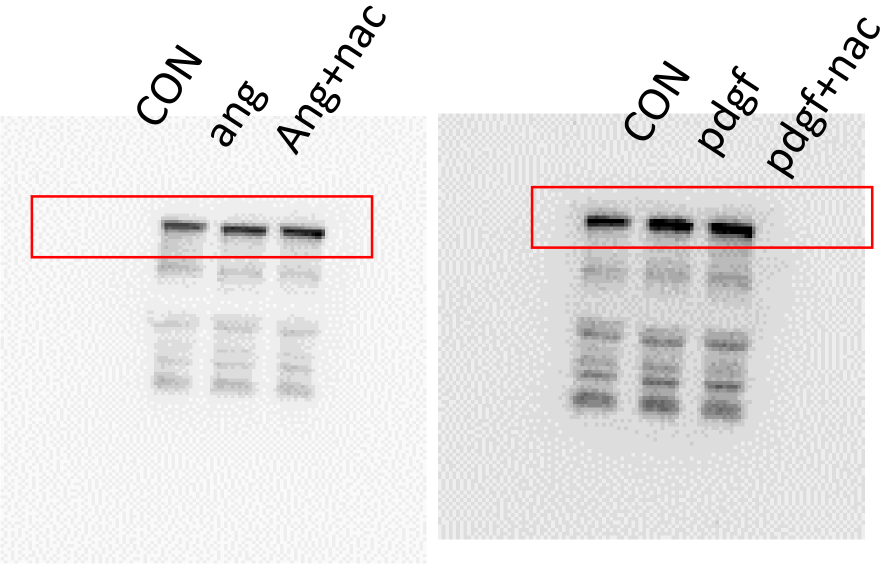


Fig.1f,g-col1A

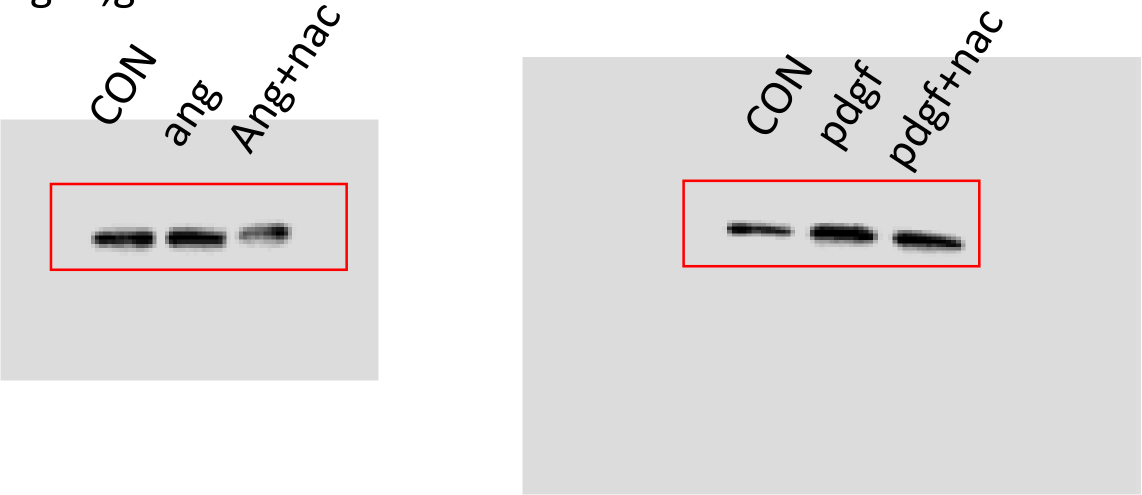


Fig.1f-actin Fig.1g-actin

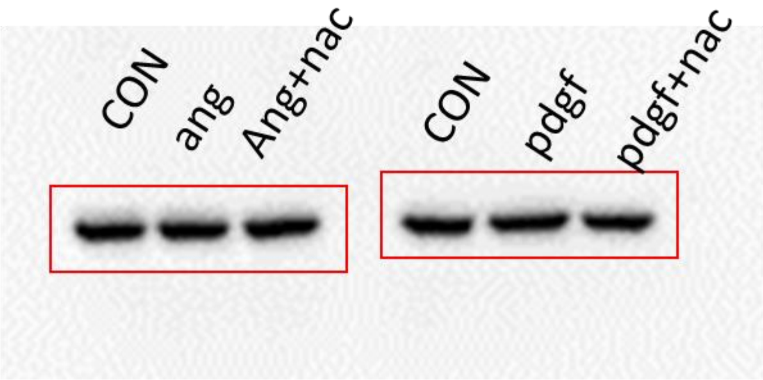

Fig.3H-COL1A

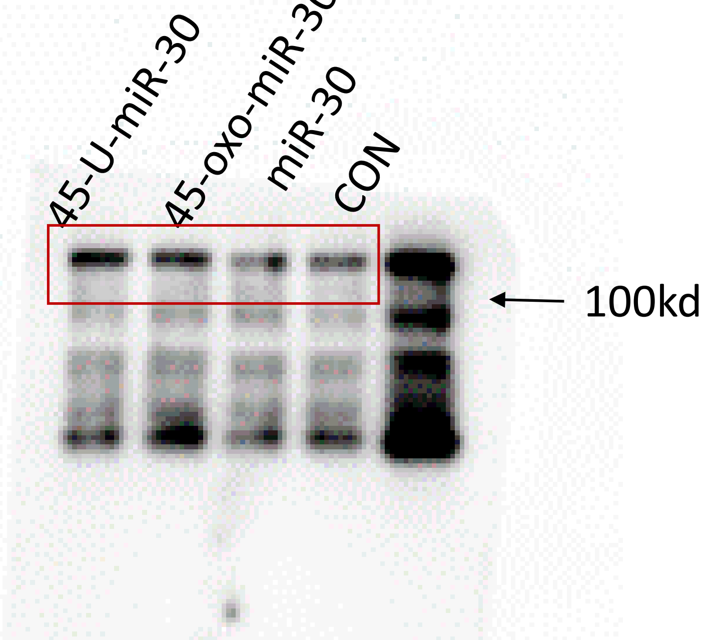


Fig.3H-actin

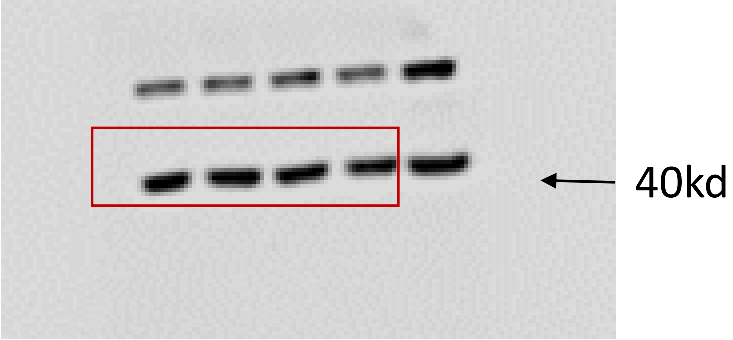


Fig.5C-CDKN2C(P18)

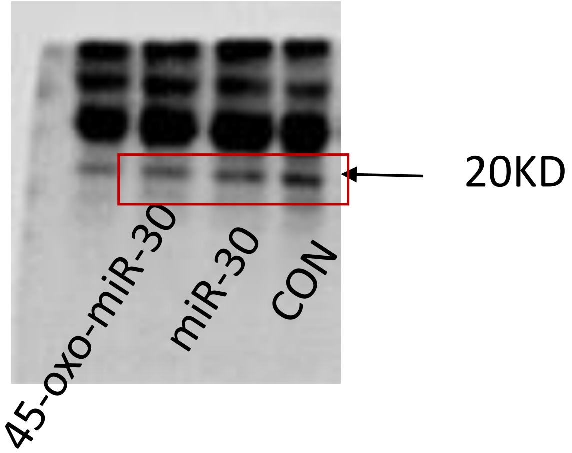


Fig.5C-actin

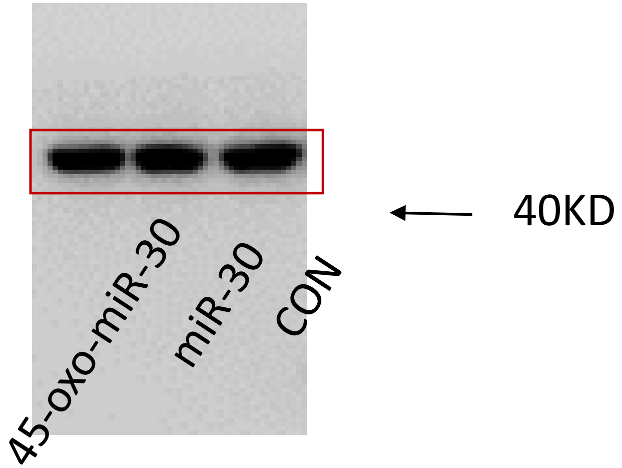

Fig.6A-CDKN2C(P18)

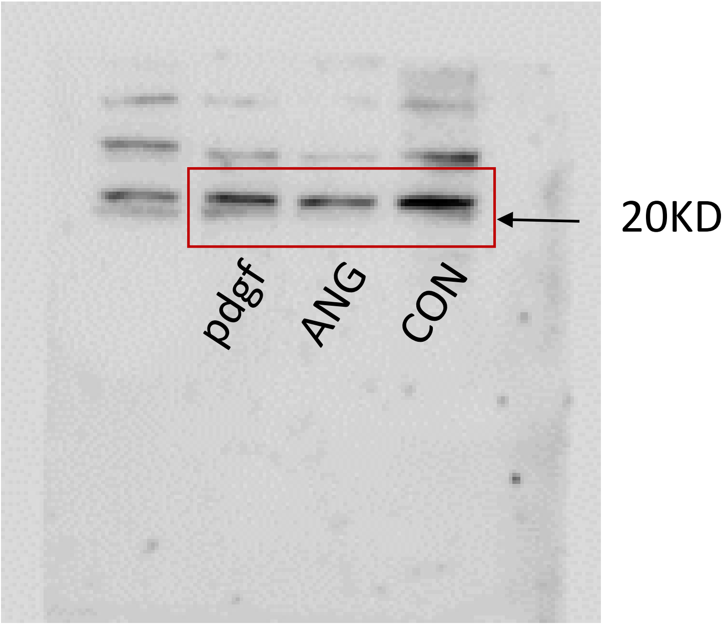


Fig.6A-actin

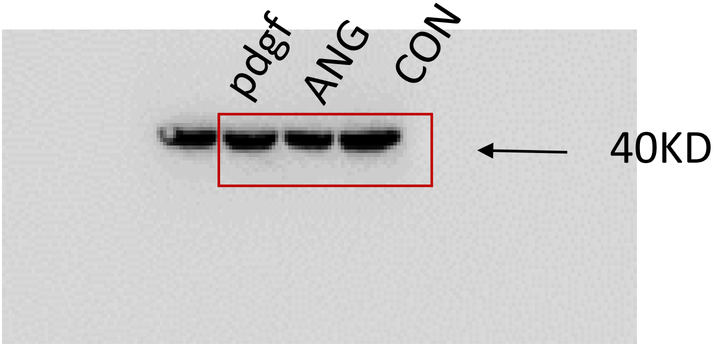

Fig.6BC-col1A

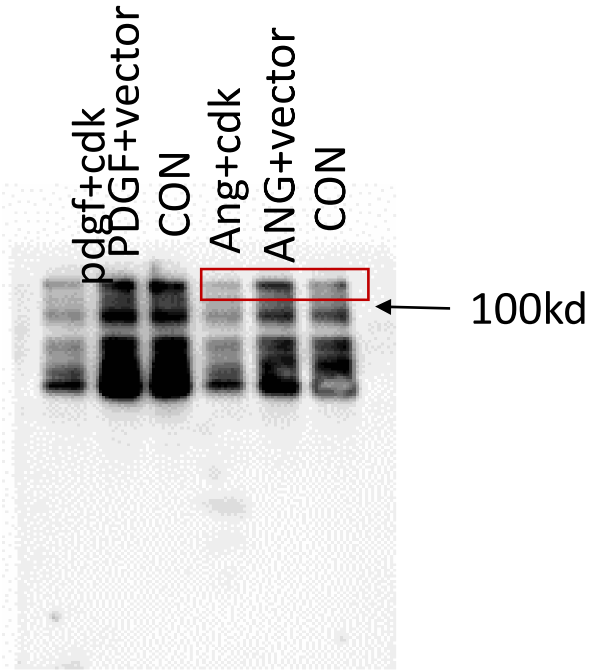


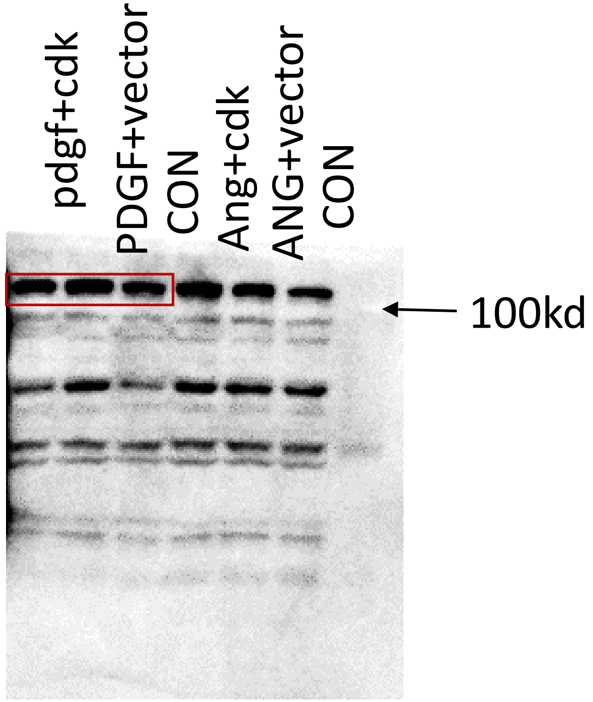


Fig.6BC-actin

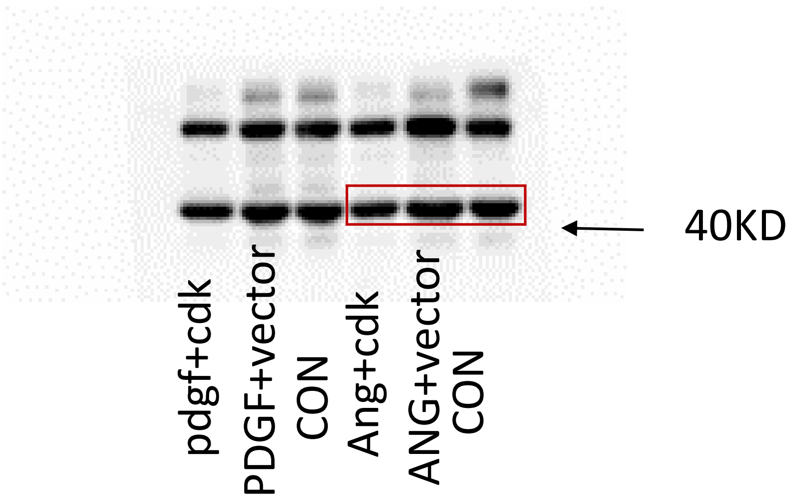


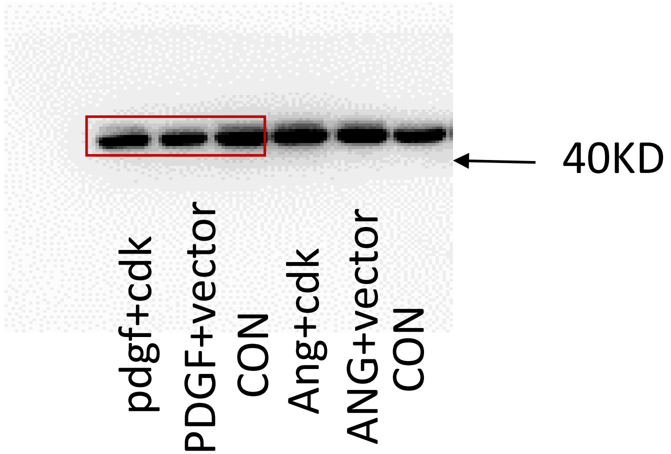

Fig.6BC-CDK4

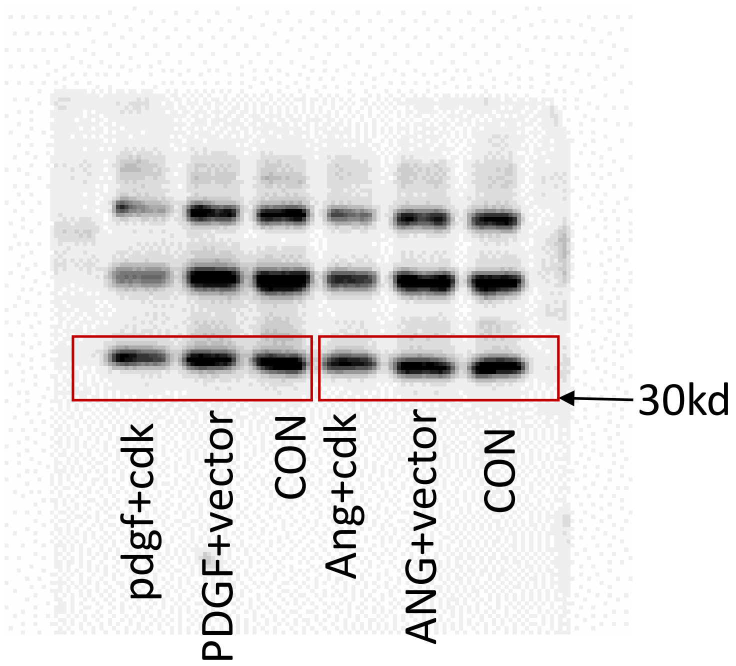

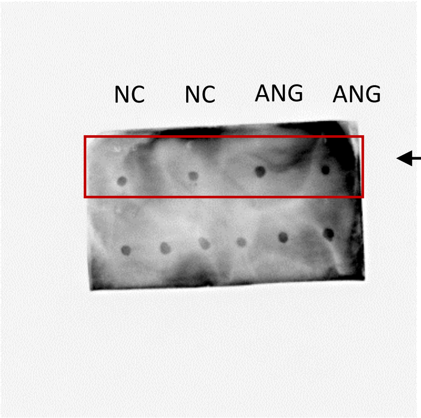


Fig.1a

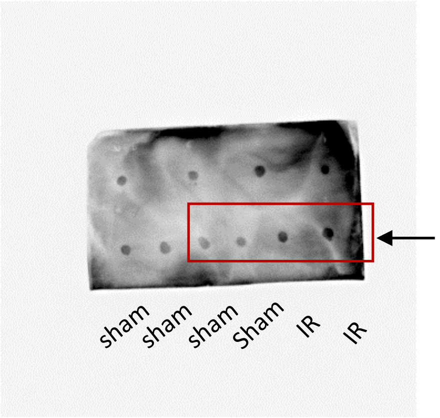


Fig.1b

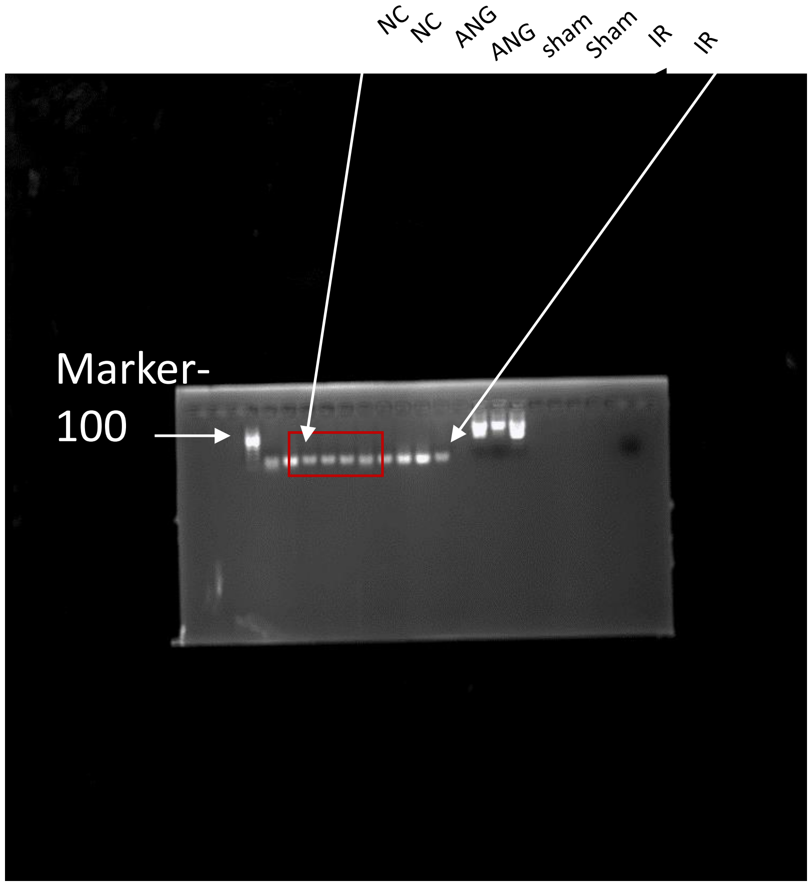


Fig.1a total miRNA

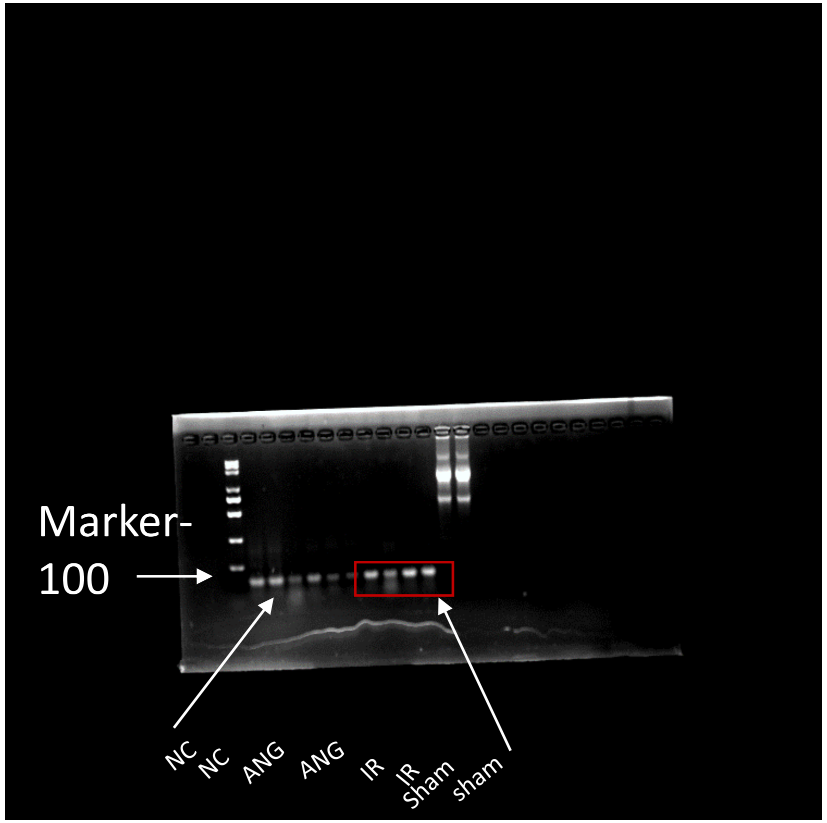


Fig.1b total miRNA

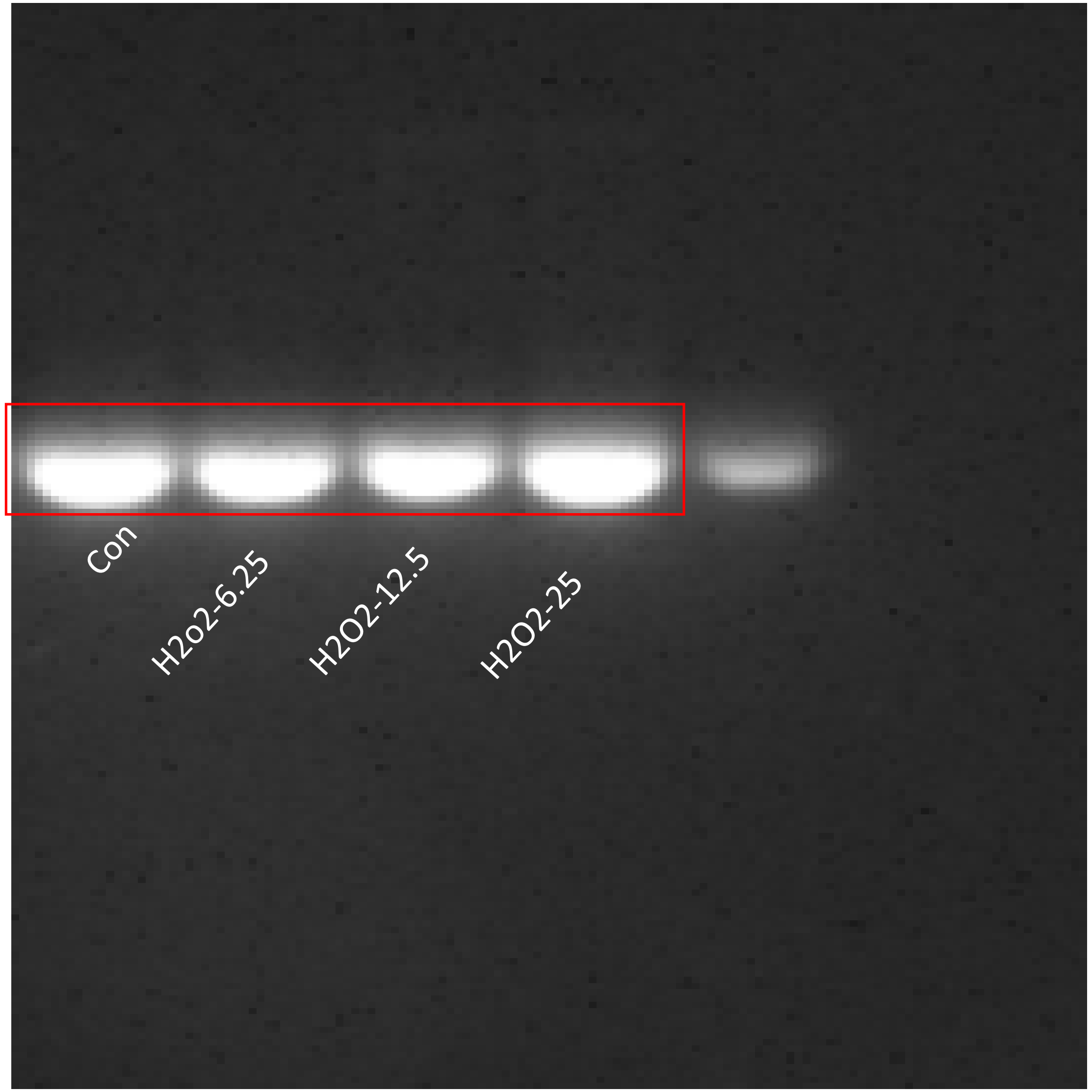


Fig. 2b-Total miRNA

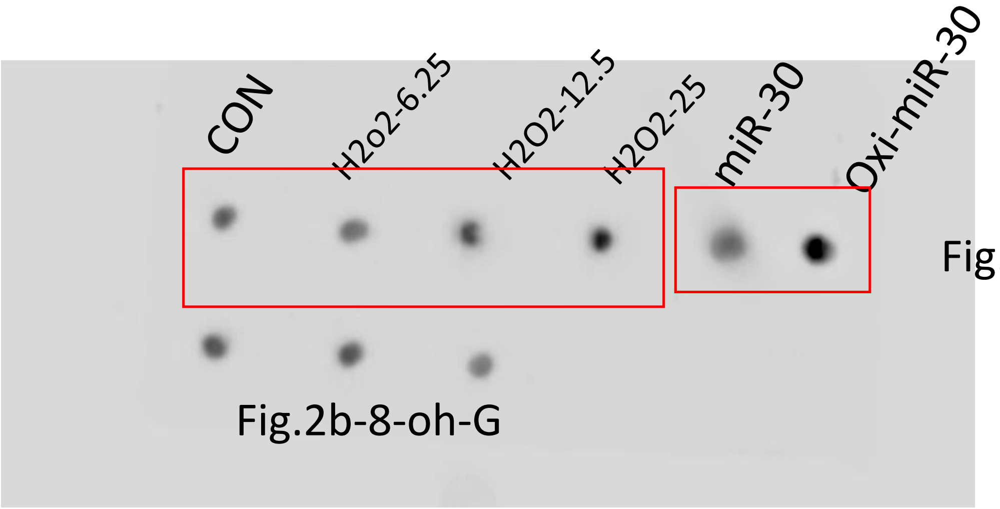


Fig.3a-8-oh-G

Fig.2b-8-oh-G

Fig.1 h-8-oh-G

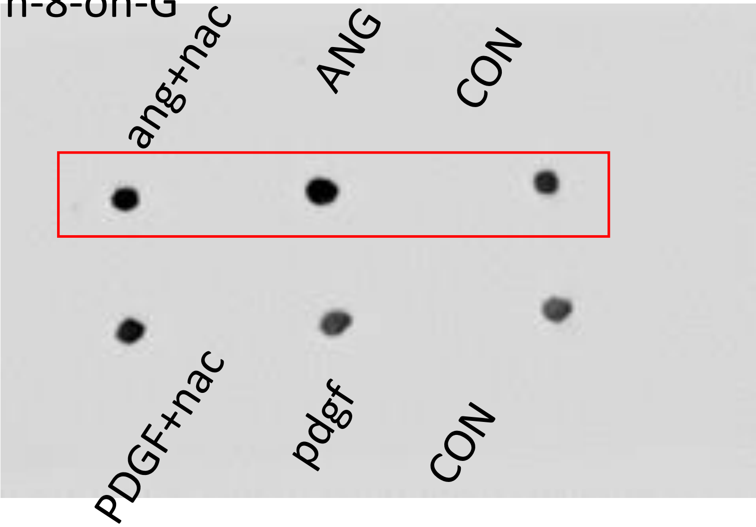


Fig.1 I-8-oh-G

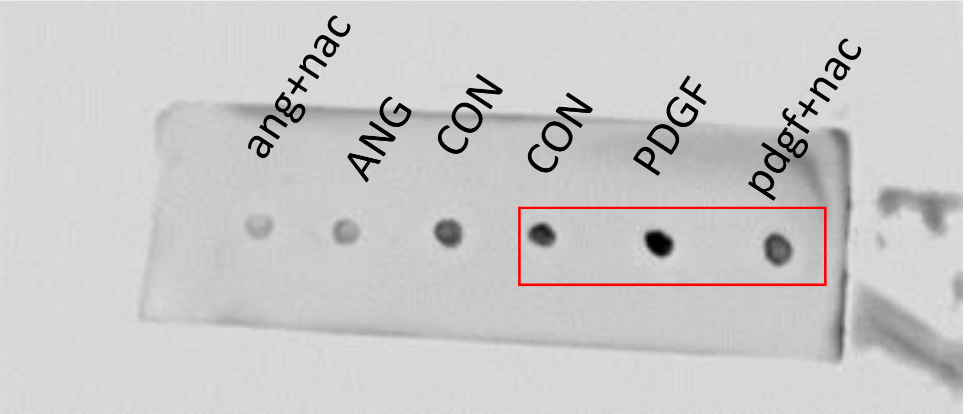


Fig.1 I-Total miRNA

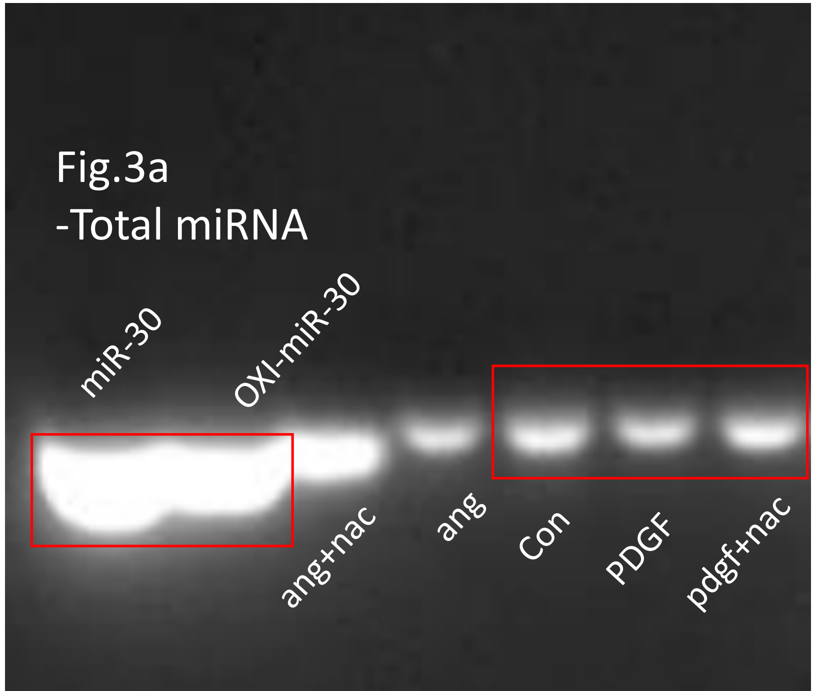


Fig.1 H-Total miRNA

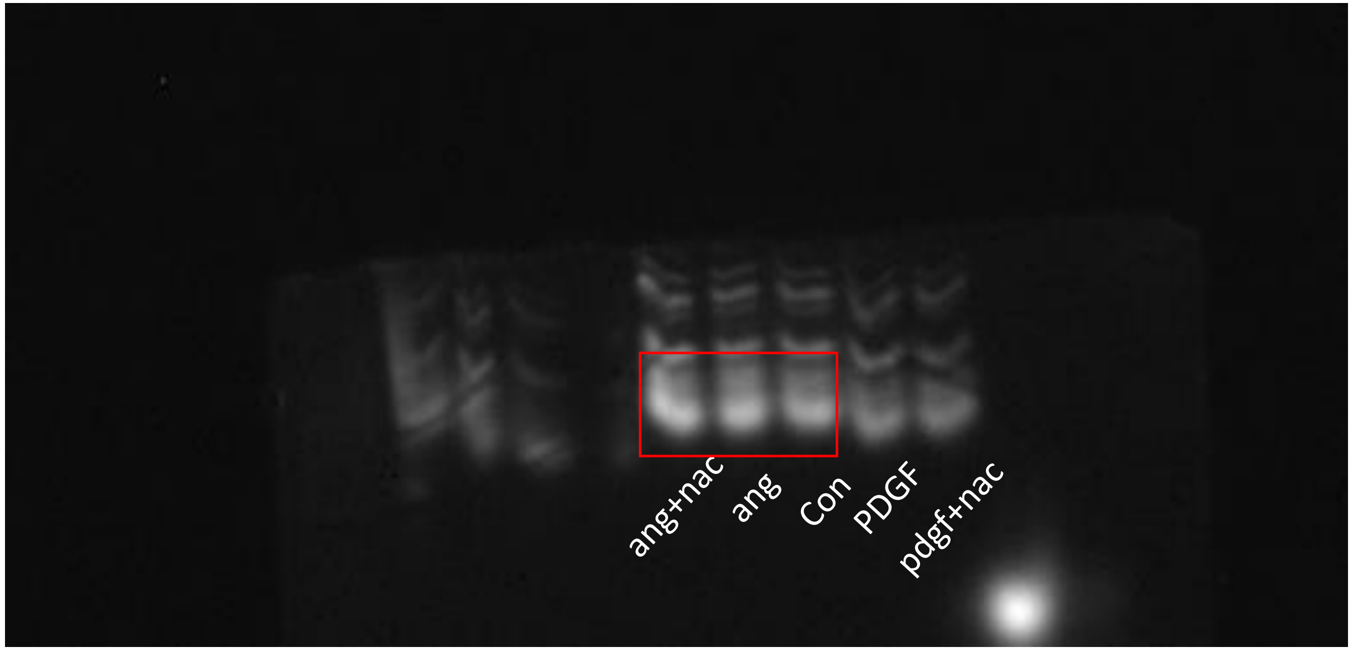

Supplement: Supplementary file 1 — Supplementary Information 1. [file 41598_2024_63635_MOESM1_ESM.pdf]
