# Supplementary figures and images for: Oxidative modification of miR-30c promotes cardiac fibroblast proliferation via CDKN2C mismatch
Source: Sci Rep. 2024 Jun 7;14:13085. doi: 10.1038/s41598-024-63635-2 (PMC11161483; doi:10.1038/s41598-024-63635-2)

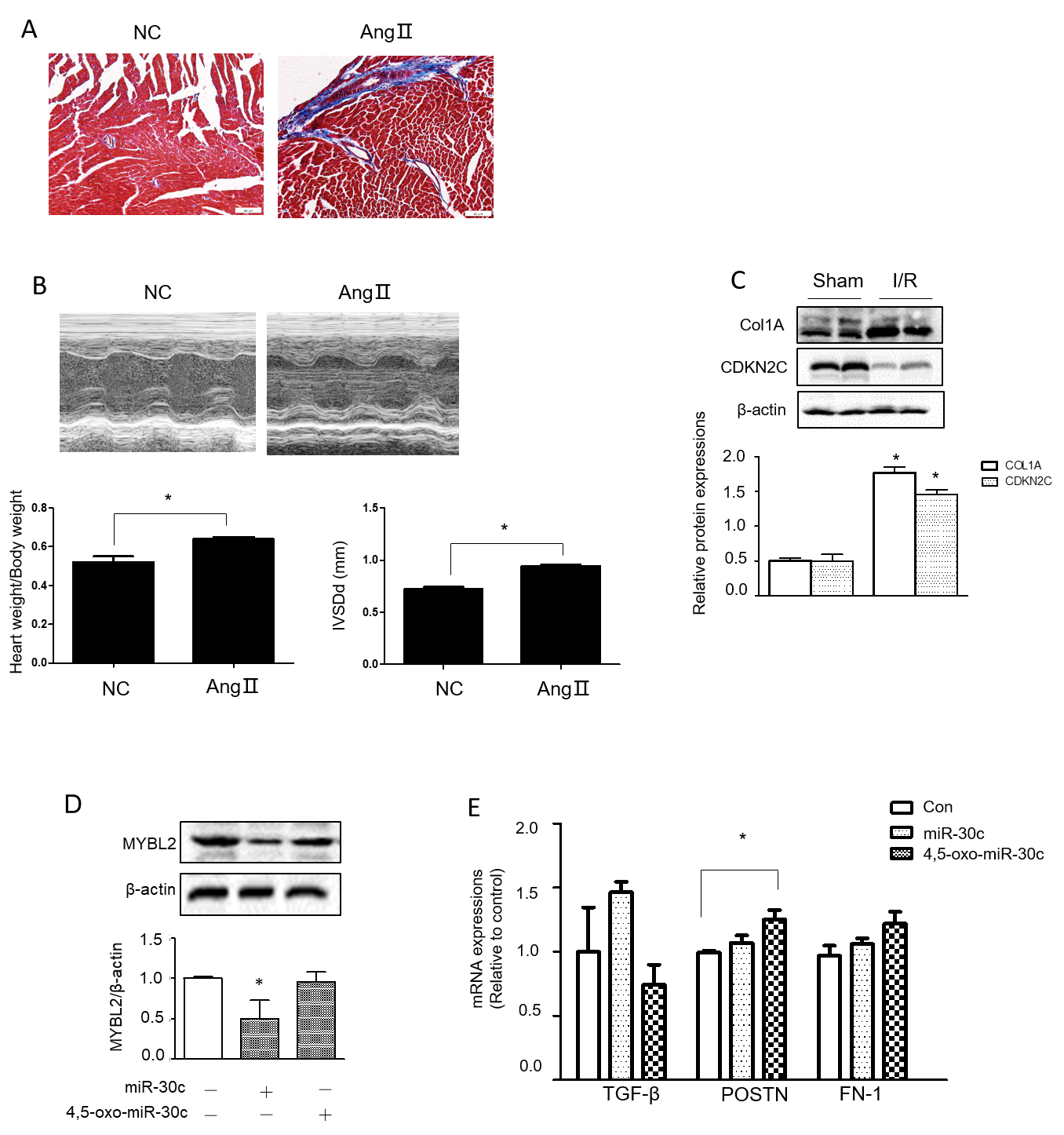

Supplement: Supplementary file 2 — Supplementary Information 2. [file 41598_2024_63635_MOESM2_ESM.tif]
