# Supplementary material for: Oxidative modification of miR-30c promotes cardiac fibroblast proliferation via CDKN2C mismatch
Source: Sci Rep. 2024 Jun 7;14:13085. doi: 10.1038/s41598-024-63635-2 (PMC11161483; doi:10.1038/s41598-024-63635-2)
Supplement: Supplementary file 3 — Supplementary Information 3. [file 41598_2024_63635_MOESM3_ESM.docx]

Supplementary Figure legend

Figure S1. Cardiac fibrosis are found in vitro and in vivo.

(A) Representative Masson staining of heart tissues from control and Ang II-induced heart injury animals. (B) Heart weight (left) and IVSDd (right) by M-mode echocardiography. (C) CDKN2C and COL1A expression in heart tissue from sham and IR mice. * p<0.05 compared to the sham group. (D) Representative Western blot (top) and densitometric analysis (bottom) of MYBL2 protein levels in miR-30c and 4,5-oxo-miR-30c NRCFs. (E) mRNA expression in cells treated with miR-30c or 4,5-oxo-miR-30c, * p<0.05 compared to the control group.
